# Appeal of the Widow of Horace Wells

**Published:** 1860-07

**Authors:** 


### ARTICLE XV.

### *Appeal of the Widow of Horace Wells.*

Hartford, *April,* 1860.

Sir :—As the widow of Dr. Horace Wells, I beg leave to address you. The discovery which my husband made, and which has so largely benefited mankind, has been to his family only a source of bitter misfortune. The experiments which he constantly made upon himself terminated fatally, and he died in fear and despair that the fame due him would not be accorded after his death.

The only inheritance which Horace Wells has left is the reputation which he had earned as a benefactor of mankind, and my highest ambition is to leave this unquestioned before the world. In pursuance of this object, it is my intention to bring this subject before the Medical Convention, to be held at New Haven during the coming summer. I feel assured that there, at least, I shall have a full and patient hearing, and that my husband's brother physicians will deliberate well before they forsake a just cause—when it is that of the widow and orphan. Although it may now be too late to do anything but justice to my husband's memory, I pray that at least this may be accomplished, and that the evidence that he is the true discoverer may be endorsed by the Medical Convention. To this end, let me beg you to give some attention to the evidence which will be forwarded to you. It has been prepared by the friends of a helpless woman, whose duty it is to redeem the memory of a good man, and rescue the credit of his discovery from the grasp of men, who, presuming upon his sensitive nature, and afterwards upon my helpless widowhood, have laid claim to a discovery which I know belongs to my husband alone.

Yours, respectfully,

ELIZABETH WELLS.

---

### "WHO CONQUERED PAIN?"

Amongst those who have reflected immortal honor on their age and country—those who are entitled to be esteemed benefactors of mankind, is Dr. Horace Wells, a name apparently so little known or so little regarded *now*, notwithstanding the priceless boon he has bestowed upon the human race, that the reflective mind marvels at the stolid ingratitude which has suffered his merits to be eclipsed, and permitted even temporary oblivion to rest upon his great achievements.

The gift thus bestowed upon humanity by Dr. Wells was so wonderful, so priceless, that had pagan Greece or Rome been so beholden to a man, he would have been elevated in their esteem to the rank of the beneficent deities; temples would have been graced with his statues, and incensed burned to signalize the great benefaction. Nay, so startling was his discovery, so far in advance of all others was the good conferred, that only amongst the fables of a heathen mythology, or in the marvelous tales of the "Arabian Nights," can parallels be found, where one deity or genius bestows beauty, another riches, another immortality, to still the ceaseless cravings and complainings of the human race.

That marvelous gift to life was "IMMUNITY FROM PAIN." Yes, the body of man, a bundle of nerves at the best, was to arrive at a period in its history when even the hacking knife and grating saw of the surgeon might be smiled upon by the patient himself as diseased limbs and flesh were cut asunder. Yet, while monarchs and learned academicians exulted over the invaluable benefit—while the "groaning" hospital relapsed into silence and repose before its benign approach, the great discoverer of the god-like boon was suffered to sink almost friendless and unregarded to a premature grave.

Can this be so? Is it true that in an enlightened age and amongst enlightened nations such a man should have been suffered to live almost unregarded and to perish comparatively unknown? Have the cries and shrieks of pain from battle fields and hospitals died out in eternal silence under the influence of this discovery, while he breathed his last in neglect and sorrow, and does no statue of the immortal benefactor grace vestibule or place? Can lying impostors and charlatans appropriate his honors, and, denying his merits, grasp undeserved rewards from the blinded multitude of even philosophers, statesmen, and men of science?

Dead, like the great philosopher, Sir Humphry Davy, as the consequence of pursuing too far his own discoveries—devoured, like the fabled Actæon, by his own hounds, let the world *now* come forward and do deserved, albeit tardy, justice to his merits and his memory.

Reminded once more of injustice to him by late accounts of military hospital practice on the great battle fields of Italy, Montebello, Magenta, and Solferino, if we would finally vindicate his claims to the memory of a public benefactor, action can no longer be delayed, because a few more years sped, and the host of unimpeachable living witnesses will be gone from the stage of action, leaving to another generation the performance of a sacred duty—a duty which fairly belongs to the present.

As a powerful synopsis of the case, then, as an introduction to the question at issue, let us first appeal to one of the most eloquent articles concerning this matter that we have ever seen on any subject. It is high authority, too, from the editorial department of a late number of the *Virginia Medical Journal*, published at the capital of that State.

We have given the foregoing in *advance* of the testimony to be adduced upon the points at issue, because the question thus clearly presented, when followed out in the same order by irrefragable evidence, becomes thereby of easy apprehension to all—even to those who have not time or inclination to examine a large mass of testimony in detail.

And, guided by the landmarks so plainly laid down, we shall endeavor, while seeking to do justice to the much regretted dead, so to exhibit the question in all its bearings as to avoid any tedious array of facts, whilst not a doubt will be left to weaken the claim of Dr. Wells to the high honor sought.

The great *leading fact* is undeniable in regard to the only other two claimants for the honor, that one (Morton) made his experiment with ether the 30th day of September, 1846, whilst the other (Jackson) claims the discovery "in

*conjunction*" with Morton, *over his own signature,* no earlier than the 26th of October, 1846.

*This is all we have to do with as against Dr. Wells.*

And this, to them, is rendered absolutely of no value, since each, attempting to cheat the other, had rendered the testimony of either worthless as to Dr. Wells. In his quarrel with Jackson, Morton declares, over his own signature, that Jackson pronounced the discovery "a humbug," and that it was reckless in him (Morton) to use it as he does ; that he (Jackson) did not care what Morton did with the discovery, "*if he does not drag my name in with it.*" Placing their assumed discovery *as late* as 26th October, 1846, in the specification of *their* JOINT *patent,* Dr. Jackson *made oath* "THAT IT HAD NOT BEEN PREVIOUSLY KNOWN." With the fact established beyond all shadow of controversy, that the time above specified was the earliest period at which either of the men claim the merit of a discovery, let us make a synopsis of the testimony in favor of the discovery made by Dr. Wells TWO YEARS *previous.*

SYNOPSIS OF TESTIMONY.

Thos. W. Kennedy, M. D., and P. B. Mignault, M. D., made oath before Hon. Josiah Quincy, Mayor of Boston, that in the fall of 1844, while attending medical lectures given by Dr. Warren, of the Massachusetts General Hospital, they were addressed by Dr. Wells on the subject of "rendering the system insensible to pain during the inhalation of exhilirating gas." Dr. C. A. Taft testifies to the same fact, and Dr. John C. Warren certifies that these gentlemen were all in attendance on his lectures at the time specified.

Daniel T. Curtis, a citizen of Boston, also testifies before the Mayor that he was present with the medical class on the occasion referred to.

S. Fuller, M. D., of Hartford, Conn., certifies to the fact that Dr. Wells had the reputation "for more than two years prior to March, 1847, in that city, of having made a discovery which enabled him and others to extract teeth without pain, by the use of exhilirating gas ; and he adds : "There is no doubt in my mind that said Wells discovered and made the first practical application of this prnciple in surgical operations."

Dr. P. W. Ellsworth declares that to his "full knowledge, nitrous oxyd gas was administered two years earlier than this, (the period claimed by Morton and Jackson,) namely, in 1844, by Wells, and that many teeth were extracted without pain under its influence, and that Wells went to Boston at that time, as I was then informed, for the purpose of introducing the gas to the attention of surgeons in that city." Dr. Ellsworth adds : "In my mind, there is not a shadow of doubt that the whole merit of the discovery of this thing rests with Wells, and with him alone."

E. E. Marcy, M. D., of Hartford, (now of New York,) testifies to operations performed by Wells, under the influence of ether, in 1844, and says :

"In conclusion, I beg leave to offer it as my opinion, *that the man who first discovered the fact that the inhalation of a gaseous substance would render the body insensible to pain during surgical operations should be entitled to all the credit or emolument which may accrue from any substances of this nature.*"

G. B. Hawley, M. D., testifies in 1847, to the fact that he "was familiar with the successful operations of Wells" in extracting teeth without pain by the aid of nitrous oxyd gas, and he alone was regarded as the author of the discovery.

John M. Riggs, surgeon dentist, of Hartford, Conn., certifies before the Mayor of that city, that in November, 1844, he was consulted by Horace Wells "as to the practicability of administering nitrous oxyd gas prior to the performance of dental or surgical operations ;" that the trial was made ; that the *first* experiment was successful, and subsequent operations continued to be so ; "that the said Wells avowed his intention to communicate his discovery to the dental and medical faculty, and in pursuance of that intention, proceeded to the city of Boston, State of Massachusetts, for that purpose, whilst he (Dr. Riggs) continued to use the gas with great success, the patients assuring him that they felt no pain."

Mylo Lee, citizen of Hartford, testifies before the Mayor of that city to having had a tooth extracted by Dr. Wells in November, 1844, while "under the influence of the nitrous oxyd gas;" that "the operation was attended with no pain whatever."

F. C. Goodrich, citizen of Hartford, also certifies before the Mayor of that city that "during the winter of 1844" he "learned that Dr. H. Wells had discovered the mode of extracting teeth *without pain;*" that it "was accomplished by administering to the persons operated upon exhilerating gas or vapor, which, it was asserted, rendered the human system insensible to pain;" that "the Doctor was most successful, extracting from me a large firmly-set bicuspid tooth without the slightest sensation of pain;" that he witnessed "the same process by Dr. Wells upon several individuals, accompanied in every instance with perfect success."

J. Gaylord Wells, citizen of Hartford, also testifies before the Mayor "that more than two years prior to this date," March 26, 1847, that being informed of the discovery of Dr. Wells, he "inhaled the exhilerating gas, and under its influence had six teeth extracted without pain." He adds: "that for more than eighteen months from the time I first submitted to this operation by the application of gas, I heard no other name mentioned as the discoverer except that of the above-named Horace Wells."

Wm. H. Burleigh, Esq., editor of the *Charter Oak* newspaper, now of New York, testifies essentially to the same facts—namely, that two years prior to March, 1847, he had two teeth extracted by Dr. Wells without the *least suffering*, "while under the influence of the gas."

Dr. Marcy subsequently testifies further. He certifies before a magistrate of Hartford, by certificate, dated December 1st, 1849, that he "was aware of the fact of Dr. Wells' visit to Boston in 1844, for the purpose of communicating his discovery to the faculty of that city. I also had an interview with Dr. Wells soon after his return from Boston, when he informed me that he had made known to Dr. C. J. Jackson and Mr. Morton the anæsthetic properties of the nitrous oxyd gas, the ether vapor, and other analogous substances. He also informed me that he had made an imperfect trial with the gas before Dr. Warren's class, but that the experiment was not satisfactory on account of the patient's getting an insufficient quantity of the gas. He further informed me that his discovery and his whole idea respecting anæsthetic agents was ridiculed by Dr. Jackson and other medical men of Boston, *but that his former pupil, Morton, swallowed this ridiculous idea greedily, and kept it down until* 1846, *when he ejected it at Washington in the form of a patented compound—mark the word, compound—called Letheon.*"

And Dr. Ellsworth, also in another certificate of subsequent date, says: "I am perfectly aware of Mr. Wells' visit to Boston for the purpose, as stated at that time, of announcing his discovery, and giving it a fuller trial at the hospital in that city, and also his dissatisfaction with the results of his visit, both as to the success of his experiment before Dr. Warren and his class, and the reception with which his idea met." He adds: "Having full information respecting the circumstances attending the birth of this discovery, and also having carefully perused the statements of Jackson and Morton, I have seen no reason to change my opinion, or in the slightest manner doubt that to Wells alone belongs the *whole* honor of first using any substance by inhalation for the mitigation of pain.

Sixteen members of the medical fraternity of Hartford—Drs. Fuller, Sumner, Rogers, Beresford, Grant, Barry, Marcy, Taft, Dodge, Ellsworth, Russell, Hawley, Hunt,

Crary, Schue, and Lee—also express their implicit reliance npon the statements made in the various certificates quoted as bearing upon the period of the discovery by Dr. Wells, and concluded their statement in the following words: "We take pleasure, also, in expressing our entire confidence in the integrity of the said Horace Wells, than whom no person in our city is more favorably known as a gentleman of honor and integrity. We know, moreover, that he has for several years past successfully devoted himself to subjects pertaining to invention and discovery."

Finally, we have the following noble and emphatic testimonial by the assembled legislative wisdom of the State of Connecticut:

"*Resolution of the General Assembly of Connecticut, of May*, 1847.

"*Whereas*, it being understood by this Assembly that Dr. Horace Wells, of Hartford, discovered, in 1844, that nitrous oxyd gas, or the vapor of ether, inhaled [by] persons, causes insensibility to pain, in amputation, or other surgical operations, which discovery has been most honorably noticed by various medical societies in London, and by the Academy of Medicine, and by the Parisian Medical Society in France, and has since been in use in England, France, and in this country: therefore,

"*Resolved by this Assembly*, That the aforesaid discovery by Doctor Wells, of Hartford, Connecticut, of the use of *nitrous oxyd gas*, or vapor of ether, in surgical operations, is of great importance to the public, and entitles the inventor to the favorable consideration of his fellow citizens, and to the high station of a public benefactor."—*Passed by the Connecticut Legislature in* 1847.

It is some consolation to know that *late* events point to a recognition of Dr. Wells by the learned world as the one *alone* entitled to the gratitude of mankind for this great discovery, and this is seen in the fact, amongst others announced, that the Hon. Truman Smith, who has hitherto, in his place in the United States Senate, defended the claims of Dr. Wells with an ability and disinterestedness beyond all praise, in a New York paper of last year, uses the following emphatic language :

"In my former communication, I stated that I had stood pretty much alone in efforts to assert the claims of Dr. Wells and the rights of his widow and child ; but I am happy to say that is to be so no longer, as will appear from the following communication, which I have recently received from the Right Rev. Bishop Brownell, ex-Chief Justice Williams, and other citizens of Hartford, of high distinction ;

HARTFORD, *Dec.* 18, 1858.

HON. TRUMAN SMITH,

DEAR SIR :—As the city of Hartford had the honor of giving birth to anæsthesia, or the use of gases and vapors, for the alleviation of pain, and believing that the claims of the late Horace Wells, as the author of that discovery, have not been brought as fully before the world as the case demands, we request you, at your convenience, to give our citizens a history of that great discovery, and the efforts made to deprive Dr. Wells of his just rights. T. C. Brownell, Th. S. Williams, Wm. W. Ellsworth, Seth Terry, B. Hudson, Harvey Seymour, James W. Bunce, T. M. Allen, S. B. Beresford, M. D., B. Rogers, M. D., P. M. Hastings, M. D., A. W. Barrows, P. W. Ellsworth."

But we need not multiply the overwhelming proofs. The language of Dr. Wells himself is definite and emphatic. After mentioning the *first* successful experiment, that on himself, he says : "This was in the fall of 1844. Being a resident of Hartford, Connecticut, I proceeded to Boston in December of the same year, in order to present my discovery to the medical faculty ; first making it known to Drs. Warren, Hayward, JACKSON, and MORTON ; the last two of whom expressed themselves in the *disbelief* that surgical operations could be performed without pain—both admitted that this *modus operandi* was entirely new to them; and these are the individuals who now claim to be the discoverers."

There is, as we have said, an emphasis in this simple statement of Dr. Wells which must convince every unprejudiced mind, the honest judgment of all men, of the shameless injustice sought to be perpetrated, and this even in the absence of the mountain of unimpeached and unimpeachable testimony we have brought forward. And these men, mark the fact, sought a *patent* for the discovery—wished to monopolize the blessings to flow from it—whilst Dr. Wells, in the publication from which we have just quoted, says, in the manner of one whose whole being overflowed with benevolence, that, whilst he was "*well assured that it was a valuable discovery—he was desirous that it should be as free as the air we breathe.*"

Why, that was the very spirit of benevolence itself, proving the discovery worthy of the source from which it emanated. No mean spirit actuated him in seeking to vindicate his claims to the high honor to which he was entitled, and mankind now, in awarding to him what is his just due, may turn to the memory of the man himself with just pride. *He is in his grave*, a victim to his great discovery, having experimented too far upon his own frail system, and for the good of mankind. No contests or awards can disturb him more; but the dishonest attempts to deprive him of his honors have aroused those who knew his worth, who will never cease battling until his claims are established.

Some stress has been attempted to be put upon a letter written by Dr. Wells, in Oct., 1846, in answer to one from Morton of a previous date, in which he cautions Dr. Morton not to dispose of certain "rights" the latter claimed to a "*compound*" he said he had discovered. This letter is fully explained by Dr. Wells in another of April, 1847, where he says:

"The letter which is there introduced with my signature was written in answer to one which I received from Dr. Morton, who represented to me that he had discovered a 'compound,' the effects of which, *as described by him*, entirely eclipsed those produced by nitrous oxyd gas or sulphurate ether; he stating that his compound would invariably produce a sound sleep, the length of which was wholly optional with the operator; that he had not made a single failure in one hundred and sixty cases, etc. He also stated that he had obtained a patent for this compound. I accordingly started for Boston to learn more of this improvement on my discovery, with which I had made him acquainted long before. While at his office I saw the (so-called) compound administered to a patient; it apparently had the same effect as the gas, which I had many times administered for the same purpose. Before I left for home, the gas was given to several other patients, *with but partial success*—at least so said the patients with whom I conversed. I then inquired about his patent, (his assumed '*rights*,') and found, to my surprise, that he had not obtained one, *nor even made an application for one*; this being done at a subsequent period, *as the date of his specifications and patent clearly show*.

*Very effectually* does this plain statement from Dr. Wells dispose of Dr. Morton's "rights" to any superior "compound" he had claimed consideration for, in his letter; and here this part of the question may safely rest.

And now a few words further as to the light in which this invaluable discovery is viewed abroad. In Dr. Ronx's Report from the Committee of Eminent Surgeons and Physicians of France, on the Prizes in Medicine and Surgery for the years 1847 and 1848, the following decisive language is held:

"A splendid service has been rendered to science and humanity in the discovery of a means nearly infallible, or at least successful in the generality of cases, of rendering man temporarily insensible to pain, with a transient perturbation only, after which all the functions return to their natural play. There have been, indeed, deplorable instances of baneful effects by anæsthetic agents, from adscititious causes, but the number of them is infinitely small, compared with the prodigious multitude of trials. There is no exaggeration in asserting that, from the time—a little more than three years since—the inhaling ether or chloroform has been introduced into the practice of physic and surgery, a hundred thousand individuals—first in America and by American surgeons, who enjoy the glory of the initiative, and then in different parts of the world—have been subjected to it ; and in this number, not more than twelve or fifteen disastrous cases can be cited. Owing to their particular situations, some of the members of your Committee—two particularly, Messrs. Velpeau and Ronx—have been called to pay a large tribute to science as it regards the employment of anæsthetic agents. Their single experience is imposing enough. Since the end of 1846, Drs. Velpeau and Ronx have each, apart, practiced etherization, properly so called, first ; then chloroformization, five hundred times at least ; a thousand or twelve hundred individuals, or more, perhaps, have been *anœsthized* by their hands or under their eyes, in order to be subjected to surgical operations more or less grave ; and they, the surgeons, have never seen the practice attended with instant death ; both doubt that it ever had a bad influence on the consequences or proper results of their operations ; they are disposed, on the contrary, to ascribe to it a favorable influence."

The *New York Journal of Medicine*, in an able article on etherization and chloroform, uses the following emphatic language in relation to Dr. Wells :

"Although he (Dr. W.) is now beyond the reach of praise or censure, we rejoice that justice will at last be done his memory, and that professional opinion is so unanimously awarding him the sole credit of introducing and establishing the existence of anæsthetic agents ;" and in another part of the same article adds: Let us hear what Dr. Warren says on this subject.

" 'In this country,' (says Dr. Warren,) 'Dr. Horace Wells, of Connecticut, made many trials of this gas in 1844. In the autumn of that year he came to Boston, and *in company with Dr. Morton*, visited me at the Medical College, for the purpose of requesting that the Medical Class should have an opportunity of hearing some remarks on THE USE OF THE NITROUS OXYD FOR THE PREVENTION OF PAIN. These remarks were actually made, and at a subsequent day a trial of the gas took place. But as I was very much occupied at the time, these occurrences made so little impression on my mind, that when, in the latter part of 1846, we were assailed in regard to Dr. Morton's first experiments for a too great facility of adopting novelties, and the facts above mentioned were brought to corroborate the charge, I was for some time not able to understand the grounds of the attack. Dr. Wells, however, in the summer of 1847, mentioned to me circumstances which recalled to my mind his visit; *and his statement was afterwards confirmed by that of Dr. Morton.* Such are the facts within my knoweledge of Dr. Wells' efforts to discover a mode of *preventing* or alleviating pain in surgical operations. It appears that he actually did prosecute his trials in Connecticut and elsewhere to such an extent, that *when the matter was investigated by the Legislature of the State, in the winter of* 1847, *his labors were thought worthy of honorable notice.*"

"And yet," says the *Journal*, "Dr. Warren claims for Boston the honor of the discovery."

That paper continues:

"Now, let it be observed that Dr. Wells has *proved* that, as early as 1844, he had performed more than twenty successful operations, while his patients were in an insensible state, under the influence of nitrous oxyd and ETHER; that he communicated the discovery of this condition, and made known these facts to Drs. Morton, Warren, etc., in the fall of 1844, namely, the discovery of an agent 'for the prevention of pain ;' for the former state in their specification (*Boston Med. and Sur. Jour.*) that this is what they claim as their discovery! We cannot refrain from expressing our conviction that Dr. Wells has been very unfairly treated, and that the time has come for awarding him the justice he so richly merited. Is it unfair to suggest, or even unreasonable to conclude, that the tragical event that ended the labors of Dr. Wells was induced in a measure by the consciousness of his own deserts, joined to an apparent unconsciousness of them by his professional brethren? It is indeed a saddening reflection, that had his discoveries, to which others who enjoyed his confidence unjustly lay claim, been duly awarded him, and duly appreciated while he lived, he now might be (among) a valued and useful member. Rarely is desert awarded or even acknowledged here; too often, as in this case, justice comes too late, and it is only when reward is useless and praise an empty sound, that the name of the *true* benefactor is heard. But from his ashes let the truth arise, and in this mournful instance we can only say,

'Palmam qui meruit ferat.' "

And now, in view of what we have presented, in view of unanswerable testimony we have adduced, we call on the members of the Medical profession *everywhere* to come forward and do long-withheld justice to the memory of Dr. Wells. Let American Physicians and Surgeons especially, who, (in the language of one of the splendid tributes from abroad we have quoted) "*enjoy the* GLORY *of the initiative*," let the American members of the profession come forward, and do simple justice to the memory of one who procured for them, at the cost of his life, the shining honor thus handsomely acknowledged by the eminent surgeons and physicians of France. *Will they do this?*

